# Prevalence of multiple non-communicable diseases risk factors among adolescents in 140 countries: A population-based study

**DOI:** 10.1016/j.eclinm.2022.101591

**Published:** 2022-08-12

**Authors:** Tuhin Biswas, Nick Townsend, M. Mamun Huda, Joemer Maravilla, Tahmina Begum, Sonia Pervin, Arpita Ghosh, Rashidul Alam Mahumud, Shariful Islam, Novera Anwar, Rukaiya Rifhat, Kerim Munir, Rajat Das Gupta, Andre M.N. Renzaho, Helda Khusun, Luh Ade Ari Wiradnyani, Tim Radel, Janeen Baxter, Lal B. Rawal, David McIntyre, Kjersti Mørkrid, Abdullah Mamun

**Affiliations:** a Institute for Social Science Research, The University of Queensland, Queensland, Australia; b ARC Centre of Excellence for Children and Families over the Life Course, The University of Queensland, Queensland, Australia; c UQ Poche Centre, University of Queensland, Queensland, Australia; d Department for Health, University of Bath, Bath BA2 7AY, UK; e Institute of Nursing, Far Eastern University, Manila, Philippines; f The George Institute for Global Health, UNSW Sydney, New Delhi, India; g NHMRC Clinical Trials Centre, Faculty of Medicine and Health, The University of Sydney, Australia; h Institute for Physical Activity and Nutrition, Faculty of Health, Deakin University, Melbourne, Victoria, Australia; i Institute of Health Economics, University of Dhaka, Dhaka, Bangladesh; j Department of Soil, Water and Environment, University of Dhaka, Dhaka-1, Bangladesh; k Developmental Medicine Center, Boston Children's Hospital, Harvard Medical School, Boston, MA, USA; l Department of Epidemiology and Biostatistics, University of South Carolina, USA; m Translational Health Research Institute, Western Sydney University, Penrith, Australia; n Maternal, Child and Adolescent Health Program, Burnet Institute, Melbourne, Australia; o SEAMEO Regional Center for Food and Nutrition (RECFON) - Pusat Kajian Gizi Regional Universitas Indonesia (PKGR UI), Jakarta, Indonesia; p School of Health, Medical and Applied Sciences, College of Science and Sustainability, Central Queensland University, Sydney Campus, Australia; q Mater Clinical Unit, The University of Queensland Brisbane, Australia; r Global Health Cluster, Division for Health Services, Norwegian Institute of Public Health, Oslo, Norway

**Keywords:** Non-communicable disease, Adolescents, Burden, Risk factors

## Abstract

**Background:**

Modifiable non-communicable disease (NCD) risk factors are becoming increasingly common among adolescents, with clustering of these risk factors in individuals of particular concern. The aim of this study was to assess global status of clustering of common modifiable NCD risk factors among adolescents.

**Methods:**

We used latest available data from nationally representative survey for 140 countries, namely the Global School-based Student Health Survey, the Health Behaviour in School-Aged Children and the longitudinal study of Australian Children. Weighted mean estimates of prevalence with corresponding 95% confidence intervals of nine NCD risk factors - physical inactivity, sedentary behaviour, insufficient fruits and vegetable consumption, carbonated soft drink consumption, fast food consumption, tobacco use, alcohol consumption and overweight/obesity - were calculated by country, region and sex.

**Findings:**

Over 487,565 adolescents, aged 11–17 years, were included in this study. According to trend analysis, prevalence of four or more NCD risk factors increased gradually over time. Prevalence of four or more NCD risk factors was 14.8% in 2003–2007 and increased to 44% in 2013–2017, an approximately three-fold increase (44.0%). Similar trends were also observed for three and two risk factors. Large variation between countries in the prevalence of adolescents with four or more risk factors was found in all regions. The country level range was higher in the South-East Asia Region (minimum Sri Lanka = 8%, maximum Myanmar = 84%) than Western Pacific Region (minimum China = 3%, maximum Niue = 72%), European Region (minimum Sweden = 13.9%, maximum Ireland = 66.0%), African Region (minimum Senegal = 0.8%, maximum Uganda = 82.1%) and Eastern Mediterranean Region (minimum Libya = 0.2%, maximum Lebanon = 80.2%). Insufficient vegetable consumption, insufficient fruit consumption and physically inactivity were three of the four most prevalent risk factors in all regions.

**Interpretation:**

Our results suggest a high prevalence of four or more NCD risk factors in adolescents globally, although variation was found between countries. Results from our study indicate that efforts to reduce adolescent NCD risk factors and the associated health burden need to be improved. These findings can assist policy makers to target the rollout of country- specific interventions.

**Funding:**

None.

**Research in context**

**Evidence before this study**

We systematically searched PubMed, EMBASE, CINHAL with a combination of MeSH heading terms and keywords. The key words used in the search (“health behaviours” OR “lifestyle risk behaviours” OR “health risk behaviours” OR “tobacco” OR smoke* OR “alcohol” OR “physical activity” OR “physical inactivity” OR “overweight” OR “obesity” OR “sedentary behaviour” OR “fruit intake” OR “vegetable intake” OR “diet”) and (“adolescents” OR “child*” OR “teenager” OR “youth”) and (“developing country” OR “low socioeconomic status” OR “low income country” OR “middle income country” OR “low- and middle-income country” OR “ high income country” OR “low and middle income to high income countries” OR “LMIC-HICs” OR “LMICs”). The literature search was conducted up to August 30, 2021. We did not find any comparative study prevalence of multiple NCD risk factors among adolescents across regions.

**Added value of this study**

This is the first study to comprehensively estimate the population level clustering of NCD risk factors among adolescents across regions. We used data from the Health Behaviour in School-aged Children (HBSC), Global School-based Student Health Survey (GSHS) and The Longitudinal Study of Australian Children (LSAC) of adolescents, aged 11–17 years, in 140 countries in the six World Bank regions to show the geographic variation in prevalence of clustering of NCD risk factors in 140 countries.

**Implications of all the available evidence**

This study shows that a large proportion of adolescents in all countries irrespective of region are exposed to clustering of NCD risk factors, though there is large variation in prevalence. More than 50% of countries have a high burden of NCD risk factors (prevalence of four or more NCD risk factors ≥ 50%). Insufficient vegetable consumption, insufficient fruit consumption and physically inactivity were three of the top four most prevalent risk factors in all regions.

Alt-text: Unlabelled box

## Introduction

A recent Global Burden of Disease study reported chronic non-communicable diseases (NCDs), such as diabetes, hypertension, cardiovascular diseases (CVDs), are the leading causes of global mortality.^1^^,^^2^ Risk factors such as poor diet, smoking, sedentary behaviour, and overweight/obesity increase the risk of NCDs, with most of these commencing in early life, impacting health throughout the life course.^3^ Among adolescents aged 10–20 years, physical inactivity and sedentary behaviour (e.g., watching television, gaming, and computer and smartphone use) have been linked with increased obesity and adiposity, poor diet (insufficient fruits and vegetable consumption, salt, sugar-sweetened beverages and saturated fat consumption, low iron etc), depression, and reduced quality of life.^4^^,^^5^ Globally, unhealthy diet mainly from the consumption of sugar-sweetened beverages and saturated fat increases the risk of CVD.^6^ NCDs during adulthood is linked with behavioural risk factors established early in life,^1^ and around two-thirds of premature deaths in adults are associated with behaviours adopted during adolescence.^3^

Most NCDs share a number of behavioural risk factors that are unlikely to occur in isolation, rather they typically cluster and interact to exponentially elevate the risks of NCDs.^7^ It is already well known that the collective impact of multiple risk factors increases NCD risk. Hence, comprehensive understanding on the clustering of adolescent NCD modifiable risk factors is essential in improving health and wellbeing in adolescence and adulthood.^7^

Prevention and control of NCDs has emerged as a global priority in the Sustainable Development Goals (target 3.4).^2^ To encourage prevention and control of NCD risk factors early in life, WHO has recommended a package of integrated approaches in school health promotion, such as the Nutrition-Friendly Schools Initiative.^8^ In addition, a number of countries, including Australia and Canada, have reformed their national strategies on preventing NCD risk factors by highlighting the importance of physical activity, sleep and proper nutrition during childhood and adolescence.^4^^,^^5^ Global health leaders have also stressed the importance of preventing and controlling NCDs at key stages of life,^10^ particularly during adolescence. Due to greater plasticity, adolescence has been regarded as a crucial time to intervene and disrupt the trajectory towards poor health in adulthood.^9^, ^10^, ^11^, ^12^ The recently formed Lancet Adolescent Commission identified tobacco use, alcohol consumption, overweight/obesity, and mental health problems as the major health risks for adolescents around the world.^13^ They recommended investment in dominant NCD-related health behaviours among adolescents as a means of preventing future disease development.

Little effort has been made to describe the clustering of NCD risk factors among adolescents, on a global scale, with the majority of studies presenting country-specific^14^^,^^15^ prevalence rates of NCD risk factors that do not account for globally. A recent study using the Global School-based student Health Survey data (GSHS) reported that 1 in 3 adolescents had lifestyle related risk factors.^16^ However, this study did not include countries of European and North American regions or Australia. Given the between- and within-region variability in adolescent health.^7^ It is important to understand regional differences in the clustering of NCD risk factors in adolescents, to inform local context-specific prevention priorities across regions.

There is a lack of emphasis on systematically analyzing such data to understand better the pattern of NCD risk factors at global and regional levels and to identify the most vulnerable countries. This study aimed: i) to determine the prevalence of NCD risk factors among adolescents in 140 high and low- and middle-income countries country with a particular focus on the clustering of risk factors. ii) identify the low and high burden countries with a particular focus on the clustering of risk factors and as well individual risk factors.

## Methods

### Data sources

We included countries with data fulfilling the following criteria: cross-sectional survey data from general or school-going adolescent population; data collected through random sampling with a sample size of at least 100 individuals and representative of a national or defined subnational population that includes information on NCD risk factors including physical inactivity, sedentary behaviour, insufficient fruits and vegetable consumtion, consumption of carbonated soft drinks, consumption of fast food, tobacco use, consumption of alcohol and overweight/obesity.

### Global school-based student health survey

This study used data from the Global School-based Student Health Survey (GSHS), which commenced in 2003. The GSHS was jointly developed by the WHO and the United States Centers for Disease Control and Prevention in collaboration with The United Nations International Children's Fund, The United Nations Educational, Scientific and Cultural Organization, and The Joint United Nations Programme on HIV and AIDS. GSHS employed a two-stage cluster sampling technique. In the first stage, the schools were selected randomly. Classes that provided a representative sample of the general population aged 12–17 years were identified within the selected schools at the second stage of sampling. GSHS collected information on a wide range of health indicators using validated questions and scales.^17^ GSHS included data from 99 countries (Supplementary Table 1).

### Health behaviour in school-aged children (HBSC)

The HBSC study was undertaken in 40 Europe and North American countries in collaboration with the WHO Regional Office for Europe. The HBSC study is a cross-national school-based survey on adoelscents' health and well-being of adolescents with data collected through self-completed questionnaires administered in classrooms. The questionnaire is administered to adolescents aged 11–15 years^18^ (Supplementary Table 1).

### The longitudinal study of Australian children (LSAC)

The LSAC began in 2004 with two diferent age cohorts of Australian children.^19^ The study collects data every two years, subject to attrition from non-response or non-contact. The presented study includes the latest wave of LSAC (wave 7) adolescents aged 16–17 years.

The GSHS, HBSC and LSAC surveys captured information on a wide range of health indicators using validated items including nutrition, physical activity, hygiene, mental health, alcohol use, tobacco use, drug use, sexual behaviours, violence/injury, and protective factors. We used data from the most recent survey of countries with repeated time point data.

We gathered multiple NCD related risk factors data from these datasets following consistent definitions for each:

**Table:** 

NCD risk factors	Definition
**Insufficient physical activity**	Physical activity was calculated according to the number of times physical activity was performed for at least 60 minutes per session in the past seven days, considering any type of physical activity that increased adolescents' heart and respiratory rate. Adolescents who performed physical activities less than 60 minutes per day in the past seven days were considered to have insufficient physically activity.^20^
**Sedentary behaviour**	Sedentary behaviour was assessed by the total screen time (sum of daily television, computer and video game time) on weekdays and weekends. Individuals who spent three or more hours per day in front of the screen were considered to be ‘sedentary’, a cut-off for total screen time used previously in studies.^21^
**Insufficient fruits and vegetable consumption**	Information was separately recorded on the number of days the respondents consumed fruits and vegetables in a typical week, and the number of servings of fruits and vegetables consumed on average per day. As recommended by WHO, the consumption of less than two servings of fruits and less than three servings of vegetables per day was classified as insufficient fruit and vegetable consumption.^20^
**Carbonated soft drink consumption**	The question ‘During the past 30 days, how many times per day did you usually drink carbonated soft drinks?’ was recoded for analysis to demonstrate any soft drink consumption. Those who responded ‘I did not drink’ or ‘<1 times/day’ were classified as ‘no consumption’ and those who said ‘1 to >5 times/day’ as were classified as ‘yes’).^22^
**Fast food consumption**	The questions ‘During the past 7 days, how many days did you eat food from a fast-food restaurant such as ... ?’ was recoded for analysis to demonstrate any fast food consumption KFC, with responses ‘0 days’ as ‘no’ and ‘1 to 7 days’ as ‘yes’.
**Tobacco use**	To assess any form of tobacco use, we considered those who currently smokes and currently uses any tobacco product such as chewing tobacco ‘on at least one day during the past 30 days before the survey’.^23^
**Alcohol consumption**	Those who reported having at least one drink containing alcohol during the past 30 days were classified as consuming alcohol.^24^
**Overweight/obesity**	International age- and sex-specific cut-points were used to define overweight and obesity from body mass index (BMI) calculated from weight and height measurements. School going children were categorized as overweight if their BMI z-score was more than one standard deviation (> +1 SD) from the median BMI for age and sex.^23^^,^^25^

### Ethics statement

In each of the participating countries, the GSHS, HBSC and LSAC received ethics approval from their respective Ministry of Education or a relevant institutional ethics review committee, or both. Only adolescents and their parents or guardians who provided written or verbal consent participated. As the current study used retrospective publicly available data, we did not require ethics approval.

### Statistical analysis

Prevalence of individual risk factors were calculated through weighting collected data to ensure samples were representative of the adolescent population of the country in which they were collected. This involved using strata and primary sampling units at the country level. Weighted mean prevalence with corresponding 95% confidence intervals (95% CIs) were then reported for each country. NCD risk factors were categorized in four groups: 1) No risk factors; 2) 1 risk factor; 3) 2 risk factors; 4) 3 risk factors and 4) 4 or more risk factors. Countries were classified as having a high burden if prevalence of four or more NCD risk factors was found in more than 50% of its adolescent population.^13^ Clustering of NCD risk factors and as well as individual risk factors were done by country income classification and region. We calculated global trends of NCD risk factors for four-year intervals: 2003–2007, 2008–2012, and 2013–2017.

### Role of the funding source

There was no funding source for this study. All authors had full access to the study data, accepted responsibility to submit the publication, and approved the final version of the manuscript.

## Results

We included data from 140 countries, representing 487,565 adolescents aged 11–17 years. Of these 140 countries, 8.2% were low-income countries, 24.4% low-middle-income countries, 21.3% upper-middle-income countries and 46.1% high income countries. Ten countries (7.1%) for which data were obtained were from the South-East Asia Region, 18 (12.8%) from the African Region, 18 (12.8%) from the Eastern Mediterranean Region, 32 (22.8%) from the region of the Americas, 21 (15.0%) Western Pacific Region countries and 41 (29.2%) from the European Region (Supplementary Table 1).

**Figure 1:**
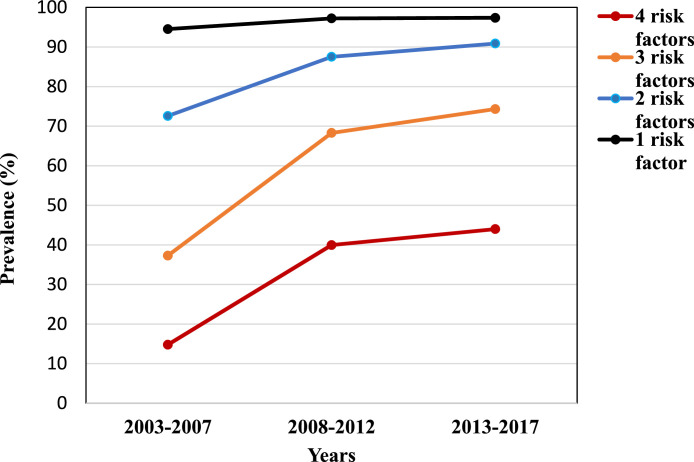
**Global trend of NCD risk factors among the adolescent.** Blue colour shows 4 risk factors, orange colour shows 3 risk factors, gray colour show 2 risk factors and yellow show the 1 risk factor.

**Figure 2:**
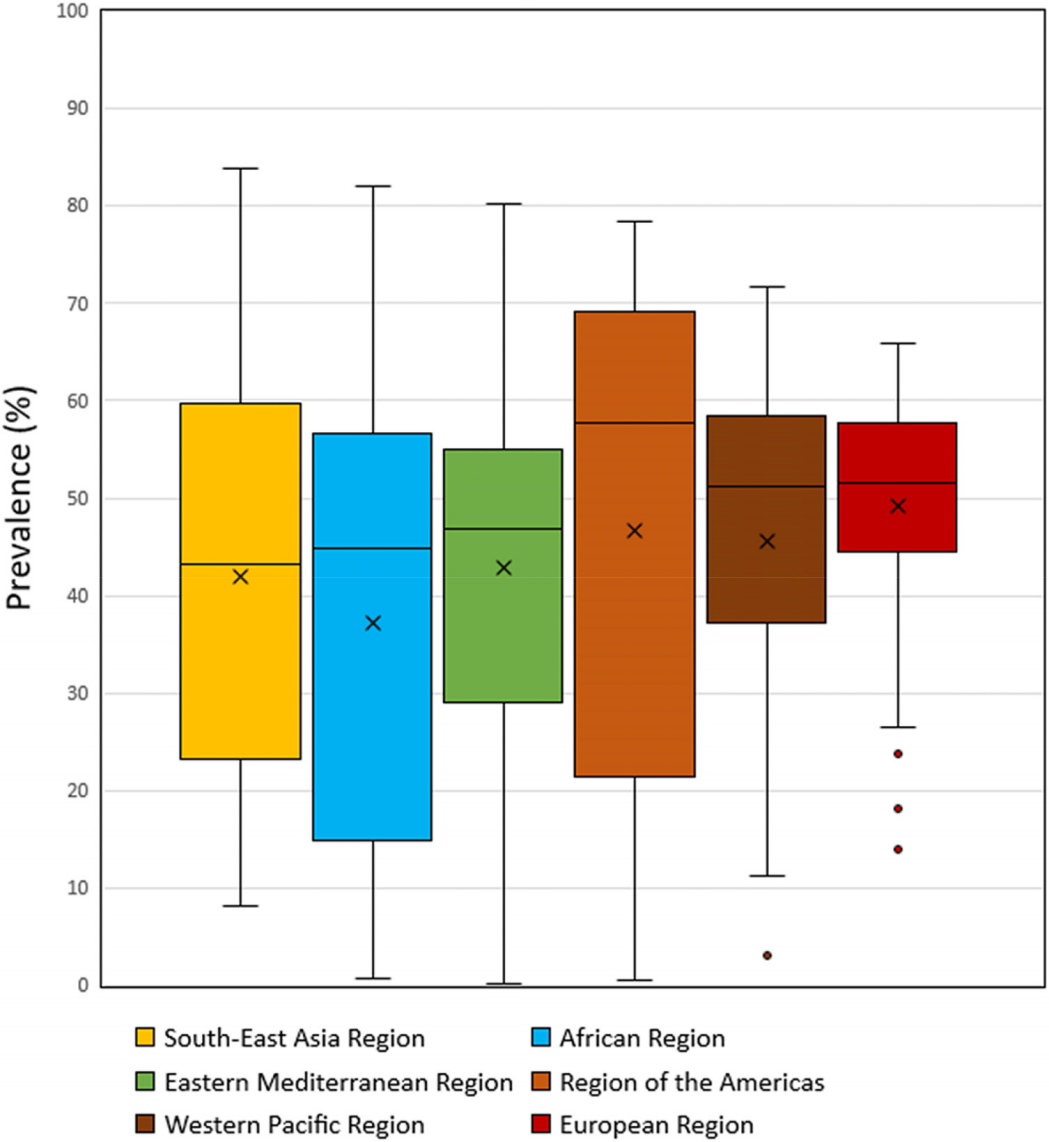
**Plots display a box representing the median, maximum and minimum percentage of four or more risk factors within region.** Blue colour show South-East Asia Region, orange colour shows African region, gray colour show Eastern Mediterranean Region and yellow show the Region of the Americas, turquoise show the Western Pacific Region and green show the European Region.

Globally, prevalence of four or more NCD risk factors increased from 14.8% in 2003–2007 to 44% in 2013-2017, an approximately three-fold increase. Similar trends were also observed for three and two risk factors (Figure 1). Large variation between countries in the prevalence of adolescents with four or more risk factors was found in all regions. The highest country level median (57.7%) was found in the Region of the Americas, although country level prevalence within the region ranged from 0.6% in Grenada to 78.4% in Curacao. The European Region (51.5%) and Western Pacific Region (51.1%) had similar medians; however, the country level range was higher in the Western Pacific Region (minimum China = 3.0%, maximum Niue = 72%) than in the European Region (minimum Sweden = 13.9%, maximum Ireland = 66.0%). Although the lowest regional level median (43.2%) was found in found in the South-East Asian Region, the highest country level prevalence was found within this region (Myanmar = 84.0%). The African Region (44.7%) and the Eastern Mediterranean Region (46.8%) had similar medians and similar ranges, (African Region, minimum Senegal = 0.8%, maximum Uganda = 82.1%; Eastern Mediterranean Region, minimum Libya = 0.2%, maximum Lebanon = 80.2%) (Figure 2). Overall, 36.4% countries have high burden of NCD risk factors (prevalence of four or more NCD risk factors≥ 50%).

**Figure 3:**
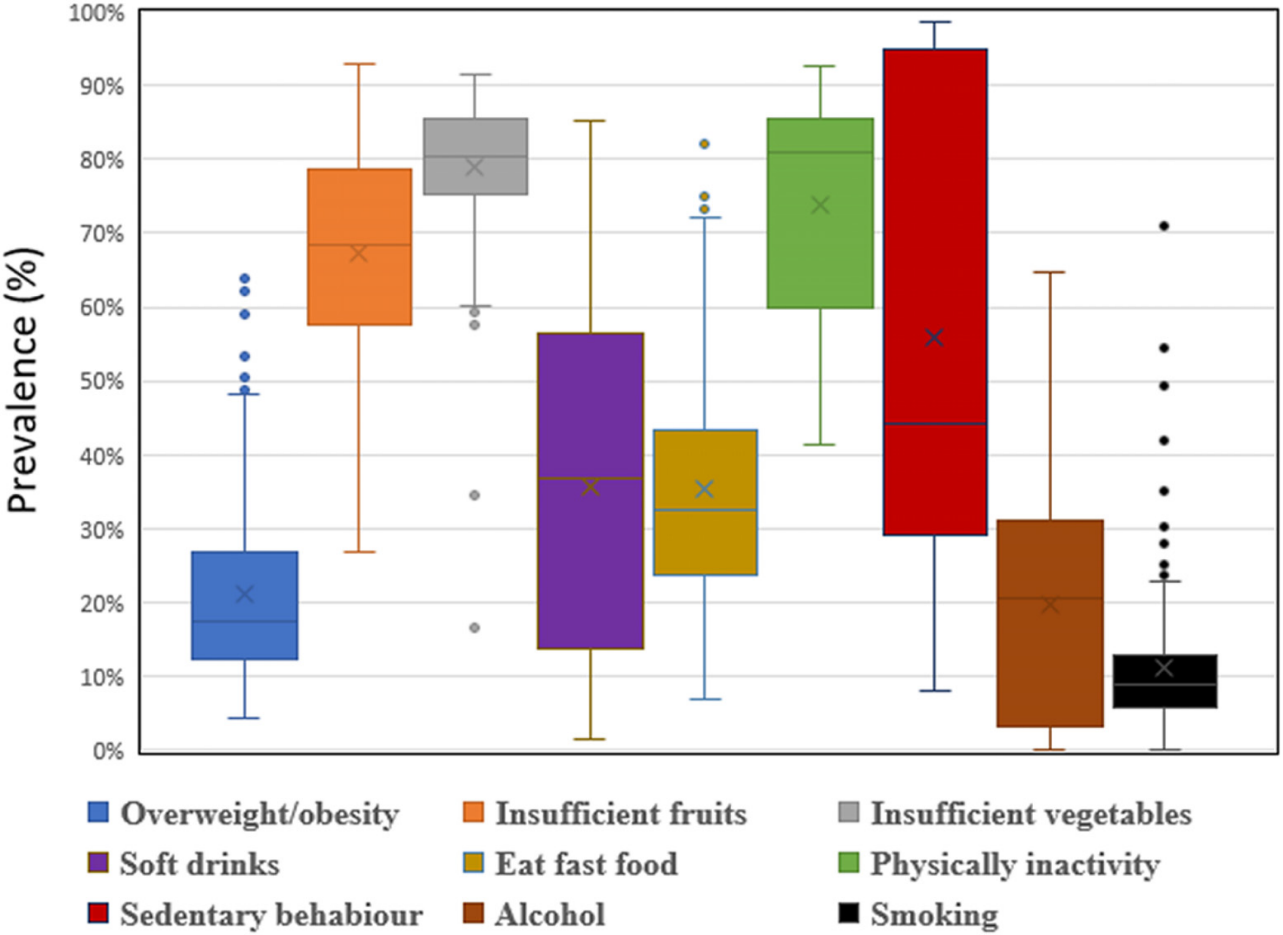
**Plots display a box representing the median, maximum and minimum percentage of individual risk factors.** Blue colour show overweight/obesity, orange colour shows insufficient fruits, gray colour show insufficient vegetables and yellow show the soft drinks, turquoise show the fast food, green show the physical inactivity, dark blue show the sedentary behaviour, brown show the alcohol and back show the smoking.

Country level median prevalence by individual risk factor was highest for insufficient vegetable consumption (86.0%), and physical inactivity (85.0%) with insufficient fruit consumption also relatively high (79.0%). Although Inter Quartile Range (IQR) was lower for insufficient vegetable consumption (11.0%) than for both physical inactivity (25.0%) and insufficient fruit consumptions (22.0%), the difference between minimum to maximum IQR was wider (min Mauritius = 27.0%, max Greenland = 93.0%). Sedentary behaviour had the fourth highest median prevalence value (44.0%) and the greatest IQR (66.0%). Tobacco use was the risk factor with the lowest country level median prevalence (9.0%) and the lowest IQR (7.0%) but ranged from a maximum in Montserrat of 49.3% to a minimum in India of 2.0%. Similarly, overweight and obesity despite having a relatively low median (21.0%) ranged from 5.8% in Viet Nam to 64.5% in Niue. Soft drinks consumption (median 37.0%, minimum Finland = 2.0% and maximum Qatar = 85%), eating fast food (median 33.0%, minimum Pakistan = 8.0% and maximum in Norway = 99.0%), and alcohol intake (median 21.0%, minimum in Bangladesh = 0.9% and maximum in Argentina = 64.9%) also demonstrated wide ranges in country level prevalence (Figure 3). Insufficient vegetable consumption, insufficient fruit consumption and physically inactivity were three of the top four most prevalent risk factors in all regions.

## Discussion

This study provides contemporary evidence on the clustering of NCD risk factors among adolescents from national, regional and global perspectives. The overall findings in this study are consistent with other multicounty studies.^16^ First, we found that prevalence of four or more NCD risk factors increased gradually over time. Secondly, variation in prevalence of clustering was found between countries within all regions. Country level prevalence clustering may reflect progress through epidemiological transitions in which mortality and fertility declined across the population structures and the dominant pattern of burden of disease. Our study revealed that overall, 36.4% countries have a high burden of NCD risk factors (prevalence of four or more risk factors in at least 50% adolescents).

One recent study by Uddin et al. using GHHS data reported that 34.9% (34.6-35.3) of adolescents had ≥3 NCD risk factors.^16^ However, this study did not include countries of European and North American regions, or Australia. Another recent study in Vietnam reported that most students had at least two risk factors and nearly a half at least three.^3^ Such clustering is concerning, it is already evident that the co-occurrence of NCD risk factors is more harmful to health than that could be projected if the individual risk factors are added independently.^26^

We found that insufficient fruit and vegetable consumption, and lack of physical activity were the most prevalent risk factors across all regions. In almost all countries, more than 40% of adolescents were physically inactive, and had insufficient fruit and vegetable consumption. Appropriate nutrition during adolescence is critical for current, future as well as intergenerational health.^27^

In this study, we found high prevalence of overweight/obesity in all regions. Data demonstrated that prevalence of overweight and obesity rises during mid-adolescence, which continues into early adulthood.^28^ This is an impending public health problem requiring further action as adolescent obesity strongly predicts adult obesity and associated morbidities. Addressing this issue at a population level will critically avert potential long-term impacts of adolescent obesity.^29^ Although the study found more favorable nutritional transition among adolescent girls and young women, there is ongoing debate as to whether gains have been as great in this younger age group.^30^ Only a few countries^33^^,^^34^ had national policies to regulate marketing of foods and beverages high in sugar, salt and fat to children. Further, most countries lack policy implementation. Some evidence suggests that additional taxes may have a ‘signalling’ effect to consumers to reduce consumption of salt and sugar beverage by increasing price.^31^^,^^32^ Adolescents and young adults have the poorest level of universal health coverage of any age group.^33^ It is interesting to note that social and structural determinants of adolescent health are largely ignored by the broader health system.^34^

It is recommended that countries implement prevention approaches that target multiple risk factors, to tackle clustering of risk factors, such approaches have been found to have a great impact at a lower cost than that of the individual risk factors prevention approaches. This means that these strategies may be particularly suitable for low-income and resources limited settings, in targeting the burden of NCD risk factors among adolescents.^35^^,^^36^ Recently, WHO and UNESCO launched a new initiative “Making Every School a Health Promoting School” through the development and promotion of Global Standards for Health Promoting Schools.^37^ School health promotion is known as an effective approach for combating NCD risk factors among adolescents. However, although health promoting school approaches are promoted, with guidance provided on their development and implementation, we found that less than half of LMICs have rigorously implemented their respective national guidelines for health-promoting schools. Lack of health-promoting schools may be related to their insufficient open space for physical activities or sports particularly in urban settings. We urge that the concerned authorities to firmly enforce these requirements as part of accreditation standards for educational institutions.

On the other hand, WHO has recommended a multisectoral approach for combating NCDs to strengthen the government's role in prevention, development of multi-sectoral public policies and legal frameworks, and health systems strengthening.^38^ For example, under health promotion in school, a whole-school approach encourage staff and all members of school communities to work collectively and collaboratively to support student learning behaviour and wellbeing within and beyond the school environment. Several systematic reviews of interventions reported that whole-school approaches increase academic achievement as well as improve student health and well-being.^39^ A study by Sharma et al. reported that school-based Health Promotion has a positive effect on vegetable consumption and feeling depressed as well as on preventing the increase in sedentary behaviours, fighting, and suicide attempt.^40^

The present study should be viewed in the context of a number of limitations. First, there is a risk of selection bias because school attendance (GSHS data) is low in LMICs and only children who attended school on the day of data collection participated in the survey. Second, the measurement of risk factors was self-reported using a single item. While self-report is an acceptable method of measuring NCD risk factors in adolescents, there is a limitation of possible shared method variance, report, recall bias and different survey. Finally, data were collected between 2003 and 2019 presenting differential period effects on prevalence estimate. However, our estimates were adjusted for period effects. Due a high degree of heterogeneity between countries from the same regions, we decided not to pool the estimates using meta-analytic methods. More research is needed to explore the diversity within regions and across countries including cohort study. The study also has a number of strengths that help us to estimate the global adolescent prevalence of NCD risk factors. First, the GSHS, HBSC and LSAC methodology used standardized and validated questionnaires. The questionnaires did not allow skip patterns in questions enabling consistency and uniformity of comparison across participant sites. Another strength is the use of survey data with large random sample sizes taken from a wide variety of international geographical and cultural settings. Finally, the analyses were inclusive of data from 140 countries.

The prevalence of four or more NCD risk factors is substantial and varies across countries. Insufficient vegetable consumption, insufficient fruit consumption, and physically inactivity were the most reported risk factors across all regions. Country-specific findings can assist policy makers to develop more strategic evidence-based interventions. Health promotion in schools may prevent risk factor clustering; however, appropriate school-based interventions require deepers understanding of complex and clustered patterns to strategically avert the burden of NCDs in adolescents.

## Contributors

All authors critically reviewed earlier versions of the draft and approved the final manuscript. TB, NT and AAM conceived the paper. TB, NT, MMH and AAM developed the analysis plan and verified the data. TB did the analysis and wrote the initial draft. JM, TBe, SP, AG, RAM, SI, NA, RR, KM, RDG, AMNR, HK, LDAW, TR, JB, LBR, DM, KM and AAM contributed to the write up and editing. All authors confirm they had full access to the study data and accept responsibility to submit the publication and have approved the final version of the manuscript.

## Data sharing statement

The analysis dataset for this specific manuscript available from the corresponding author upon request.

## Declaration of interests

All authors declare no competing interests.

## Appendix Supplementary materials

Image, application 1
